# Malnutrition is common in children with cerebral palsy in Saudi Arabia – a cross-sectional clinical observational study

**DOI:** 10.1186/s12883-019-1553-6

**Published:** 2019-12-10

**Authors:** Abdul Rahman Almuneef, Ali Almajwal, Iftikhar Alam, Mahmoud Abulmeaty, Bader Al Bader, Mohamed Farouq Badr, May Almuammar, Suhail Razak

**Affiliations:** 1 Sultan Bin Abdulaziz Humanitarian City, P.O. Box: 10219, Riyadh, KSA 11433 Saudi Arabia; 2 Clinical Nutrition Program, Department of Community Health Sciences, College of Applied Medical sciences. King Saud University, Riyadh, Saudi Arabia

**Keywords:** Cerebral palsy, Nutritional status, Dietary intake, Growth, Saudi Arabia

## Abstract

**Background:**

Cerebral palsy (CP) is considered as the main cause of severe physical impairment and malnutrition in children. This cross-sectional study intended to survey the nutritional status of children cerebral palsy in Riyadh, Saudi Arabia.

**Methods:**

We examined 74 children (age: 1–10 yrs) with CP, who attended Sultan Bin Abdulaziz Humanitarian City (SBAHC), Riyadh Saudi Arabia. Data on age, general demographics, nutritional status, and dietary intake were collected. A child was considered underweight, wasted, stunted or thin if the standard deviation scores for his/her weight for age, weight for height, height for age and body mass index for age were ≤ −2.0 standard deviation (SD) using WHO growth standards. Multivariable logistic regression identified the factors associated with nutritional indicators.

**Results:**

More than half (56.4%) of the children with cerebral palsy were malnourished as they had z-score below <−2 SD in at least one of the four indicators. Thinness (50%) was the most common form of malnutrition, followed by underweight, stunting, and wasting. Arm anthropometrics gave similar results on the percent number of malnourished children. Factors that were independently associated with malnutrition with an adjusted OR (aOR) were as follow: age ≤ 5 yrs. (aOR: 4.29); presence of cognitive impairment (aOR: 4.13); presence of anemia (aOR: 3.41) and inadequate energy intake (aOR: 4.86) (p, for all trends <0.05).

**Conclusion:**

Children with cerebral palsy of the current study have impaired growth and nutritional status as assessed by all four common nutritional status indicators. Further large-scale community-based studies for in-depth evaluation of nutritional status and growth patterns in children with CP are needed.

## Background

Cerebral palsy (CP) has been recognized as one of the major causes of physical disability in children worldwide [1–3]. There may be variations in the prevalence of CP in children mainly because of the inconsistency of definition and classification that generate seemingly controversial results and conclusions or because of the methodological variations [4]. Nevertheless, the associated nutritional problems resulting from the disorder can be visualized to be serious both in severity and scope [3]. Impairment of sensation in cerebral palsy stays for the whole lifespan [5], therefore, related issues, importantly nutritional, are likely to sustain throughout the life if not addressed promptly.

Nutritional status is significantly affected in children with CP [1, 2, 6, 7]. The associated comorbidities include growth abnormalities, feeding complications, communication disorders, mental obstruction, seizure disorders, and auditory and visual deficiency [8, 9]. With the diminished motor and mental capabilities, the patient is unable to eat properly and is very likely to have suboptimal energy intake. Investigating a possible relation between the poor dietary intake and the presence of feeding dysfunction in CP children has been one of the prime interests of most investigators in this field [10].

Most of the studies done in developed countries show that factors like inadequate nutrient intake, feeding problems, and motor dysfunctions are associated with poor nutritional status in CP children [1, 2, 11–13]. No data, however, currently exist that report nutritional status of CP children in Saudi Arabia despite a high prevalence of this disorder in children in this country [14]. Neither, there are any studies that address the contributing factors for malnutrition in CP children in Saudi Arabia. Worldwide, relatively fewer studies authenticating the importance of nutrition in CP children are existing and these are essentially missing for Saudi Arabia. Our hypothesis is that Saudi children with CP would demonstrate poorer nutritional status compared to otherwise normal children. Therefore, we aimed in this study to examine nutritional status and establish factors that have a potential association with nutritional status of children with CP attending a care and rehabilitation clinic in Riyadh Saudi Arabia.

## Methods

### Study settings

The study was conducted in Sultan Bin Abdulaziz Humanitarian City (SBAHC), Riyadh Saudi Arabia in 2015. SBAHC is 400-bedded medical and rehabilitation center that caters medical and rehabilitation care outpatients and inpatients. Having an interdisciplinary approach and an integrated organizational structure, the patients are treated in a more specialized manner according to patients’ needs and disabilities.

### Ethical approval

The study design for the current study was approved by the Research Ethics Committee at the College of Applied Medical Science, King Saud University, Riyadh, Saudi Arabia (CAM525/3334). Written consents for participation in the study were obtained from the parents or guardians of children with CP prior to the commencement of the study.

### Location, size, and selection of the sample

Children with CP, aged 1–12 years admitted to SBAHC, who met the CP diagnosis criteria [15–17], were consecutively recruited from children visiting the CP clinic. Briefly, children diagnosed CP on the basis of their gross motor function, brain imaging and/or cranial ultrasound supported by additional tests (vision, hearing, speech, intellect, development, movement, etc) by the specialized doctors were included. These diagnosis parameters are part of routine practice of SBAHC. Given the limitations of time and resources, the study used a convenience sample and recruited children with CP admitted between January 2015 to August 2015. Initially, 101 children met the eligibility criteria, but consent was only obtained from the caregivers of 80 children. Final data could be completed on 74 children as 6 children dropped out due to various reasons mainly because most (5 out of 6) of them had a documented medical history of low birth weight (<2.5 Kg), while one child was dropped because of premature birth. Information about the socio-demographic and clinical characteristics of the study population is presented in Table 1.

**Table 1:** Demographic and Clinical characteristics of Children Cerebral Palsy (*n* = 74)

Characteristics	Number *n* = 74	Malnourished *n* = 41 (55.4%)	Normal Nutrition *n* = 33 (54.6%)
Sex of child, N (%)			
Male	44 (59)	23 (31)	21 (28)
Female	30 (41)	18 (24)	12 (16)
Age of Child, N (%)			
< 5 yrs	48 (65)	24 (320)	24 (320)
> 5 yrs	26 (35)	17 (23)	9 (12)
Severity of disease according to GMFCS levels, N (%)			
GMFCS level I-III	22 (30)	9 (12)	13 (18)
GMFCS-II GMFCS levl IV-V	52 (70)	32 (43)	20 (27)
Nutrient Intake, N (%)			
Adequate energy intake	53 (72)	18 (25)	35 (47)
Adequate Protein intake	49 (66)	17 (23)	32 (43)
Mean energy intake (% of RDA)	71.3% ± 21.1	63,2% ±26.2	78.5 ± 17.8
Mean protein intake (% of RDA)	73.2% ± 34.2	55.7 ± 33.6	90.3 ± 35.7
Feeding problems of child N (%)			
Yes	57 (77)	32 (43)	25 (34)
No	17 (23)	9 (12)	8 (11)
Appetite of child N (%)			
Poor	50 (68)	31 (42)	19 (26)
Fair/Good	24 (32)	10 (14)	14 (19)
Presence of Cognitive Impairment in child N (%)			
Yes	54 (73)	35 (47)	19 (26)
No	20 (27)	6 (8)	14 (19)
Presence of anemia N (%)			
Yes	32 (43)	23 (31)	9 (12)
No	42(57)	18 (24)	24 (33)
Creatinine			
N (%) within normal range	56 (76)	30 (41)	26 (35)
N (%) within abnormal range	18 (24)	11 (15)	7 (9)
Hematocrit			
N (%) within normal range	49 (66)	21 (28)	28 (38)
% within abnormal range	25 (34)	20 (27)	5 (8)
Red Blood Cells			
N (%) within normal range	52 (70)	21 (31)	29 (39)
N () within abnormal range	22 (30)	21 (29)	1 (1)

*GMFCS* Gross Motor Function Classification System

### Study design

In this cross-sectional study, all assessment was made in three steps as reported elsewhere [8]. Briefly, all children were diagnosed and confirmed as ‘cerebral palsy’ by a developmental pediatrician and/or neurologist at the center.

In step 1, a ‘Ten Question Screen’ was used for initial screening [13]. For the purpose of this study, CP was defined as: “[15]. In the second step, a physiotherapist further assessed the patients following the decision tree for identification of cerebral palsy [14]. In the third and final step, the developmental pediatrician and/or neurologist confirmed CP and classified it using the Gross Motor Function Classification System (GMFCS) level I-V as mentioned elsewhere [18]. Briefly, GMFCS is a validated 5-level scale that classifies the severity of motor impairment in children with cerebral palsy. Gross motor functions are based on developmental milestones, and the items of the scale are distributed among 5 dimensions (A = lying, rolling; B = sitting; C = crawling, kneeling; D = standing; E = walking, running, and jumping). As a routine practice of SBAHC, Gross Motor Function Measure (GMFM-88) tool, which is the original tool used in routine practice of SBAHC, was used for the assessment. The GMFCS level was classified by the child’s physiotherapist under the supervision of pediatrician. The medical records of the participants eligible for the study demonstrated the diagnoses of CP had been made when the patient was more than 6 months of age and all required diagnoses relating to mental retardation or other developmental delays were primarily completed by the school personnel or the developmental pediatricians.

The exclusion criteria were not to consider those children with CP who had a history of low birth weight or premature birth, metabolic, genetic, or neurodegenerative diseases or with any medical infections known to affect nutritional and growth status in any way. Also, children with CP fed through enteral or parenteral routes or children with CP receiving corticosteroids or other medication/drugs known to have an influence on growth were excluded [7].

#### Data collection

Data were collected in the following distinct stages [8]:

1. ***Structured Interviews.*** The caregivers were interviewed using a well-structured questionnaire. Questions were included to get information on diet intake, feeding problems and other associated factors that could contribute to nutritional status [7].
2. ***Physical and Cognitive Examination.*** Gross and fine motor functions were assessed based on the child’s ability to sit, grasp and on self-initiated walking and fine motor function [18]. Cognitive impairment assessment was performed as a routine clinical practice. Data on cognitive assessment were collected from the patients’ hospital files. In general, all children were assessed for cognitive impairment by a neurologist on a Mini-Mental State Examination (MMSE) adapted and validated by Jain and Passi [19] for children on the basis of a system of scores for cognitive impairments. This instrument assesses the mental functions of language, temporal and spatial orientations, attention, constructive praxis and memory [20]. The test comprehensively assesses the visual/verbal attention and visual/verbal short/long term memory. Jain and Passi [19] defined a cutoff point for cognitive deficit of two standard deviations below the mean.
3. ***Anthropometric Measurements.*** Anthropometric measurements included weight, height, arm circumference (AC), arm muscle circumference (AMC), and triceps skinfold thickness (TST). Weight (kg) and height (cm) were measured as reported elsewhere [8]. Weight was recorded on Scale-Tronix model 2002 single scale (Dynamic Scales, Inc. 1466 South 8th Street Terre Haute, IN 47802, USA). For children who could stand easily and erectly, the height was measured with a scale with a fixed and attached stadiometer (SECA 789, Hamburg, Germany). In case it was not possible or difficult to measure the standing height because of the inherent contractures or scoliosis, height was estimated from the knee-height using available authenticated equations [6]:
  $$ Height\ (cm)=\left(2.69\ast knee\ height\ in\  cm\right)+24.2 $$
  The procedure for measuring knee-height was completed while the patient was in the supine position. The common stretchable measuring tape was used for the measurement. All measurements were done in triplicate and the average was considered. All measurements were done in the morning by the same experienced examiner.
  AC was measured using a non-elastic measuring tape. TST was measured with a skinfold caliper (Lange®^;^ Power Systems, Inc., Tennessee, USA). AMC was calculated using an equation [21] as:
  $$ AMC\ (cm)= AC\ (cm)-\pi\ \left[ TST\ (mm)\right] $$
  All measurements were done in duplicate by the same person and the mean values were used for final analysis.
4. ***Dietary.*** Dietary data were obtained by 3-days dietary records, kept by parents/caregivers or nurses of children with cerebral palsy. Prior to data collection, the parents/caregivers or nurses were educated on how to measure food quantities according to the food materials that were used in the diet clinic. Nutrients were calculated from the contents of the 3-day food diaries using the Arab food analysis program (HBCN; health balance for clinical nutrition, Riyadh, Saudi Arabia) diet analysis software. The calorie and protein intake of children with cerebral palsy were used for assessment of nutrient-specific and percentage of nutrient intake based on the WHO estimated average intake as reference (22).
5. ***Assessment of feeding problems of children with CP.*** A speech and swallowing therapist assessed the feeding problems of CP children. Questions were also asked to know feeding problems (yes/no) and appetite status of the children (normal/fair vs. poor) from the parents/caregivers. For this purpose, a questionnaire, based on recommendations from previously published reports [8–13] was designed in such a way to identify the most common feeding problems including inability to self-feed, inadequate/absence of tongue lateralization, chewing problem, swallowing problem, cough/choking during feed, drooling, hypertonic tongue, inability to take solid food, constipation, sucking problem, vomiting/regurgitation, non-closure of lips around spoon, Inappropriate wide mouth opening and cry/extensor dystonia during feeding.
6. ***Laboratory Investigations.*** Under aseptic conditions, 5 ml blood was drawn and serum creatinine was quantified using the ethylene-diamine-tetraacetic acid tubes. Hemoglobin, red blood cells (RBC) and hematocrit were quantified using gel tubes. All parameters were measured using routine laboratory procedures. Normal ranges for Hb, creatinine, RBC, and hematocrit were used to classify the children ‘within normal range’ and ‘within abnormal range’ (see Additional file 1).

### Data handling

BMI was calculated from the weight and height of the patient (BMI = weight [kg]/height [m^2^]). Weight, height, and BMI values were used to calculate the sex and age normalized z-scores using the reference data for normal and healthy children [22]. Sex and age normalized z-scores were calculated using WHO Anthro- (2010) and AnthroPlus (2009). Children with extreme z-scores were excluded from the final calculations (8). The weight-for-age z-score was only calculated for children up to 10 years of age and the weight-for-height z-score for those up to 5 years of age [8]. The nutritional status of each child was assessed on the basis of z-scores. For the purpose of interpretation, a weight-for-age z-score < −2 was defined as underweight; height-for-age z-score < −2 was defined as ‘stunting’; weight-for-height z-score < −2 was defined as ‘wasting’ whereas BMI-for-age z-score < − 2 was defined as ‘thinness’ as previously reported [3, 8]. Classification of nutritional status was also performed using arm anthropometrics, i.e. AC, AMC, and TSF. Reference values proposed by Frisancho [23] (for children older than 1 year of age) and Jelliffe [24] (for those younger than 1 year) were used. Definitions of anthropometric indicators and normal ranges for Hb, creatinine, RBC, and hematocrit are summarized in additional file (see Additional file 1). Adequate energy and protein intakes were evaluated according to the Dietary Reference Intake using the Reference values of WHO.

### Statistical analysis

Statistical Package for Social Sciences database (IBM, SPSS, version 23, USA) was used for the analyses of the data that were expressed as percentages and mean (SD). Pearson’s correlation coefficient was calculated to determine any association between the factors considered and the dependent variables of stunting, underweight, wasting and thinness with *p*-value ≤0.05 as significant. The co-occurrence of more than two indicators of nutritional status in a single child was depicted using a Venn diagram and consequent correlation between nutritional indicators was calculated based on Venn diagram as reported previously [8]. Factors such as age and type of cerebral palsy were categorized, and the adjusted odds ratios (aORs) with corresponding 95% confidence intervals (CIs) were computed. A p-value <0.05 was considered significant.

## Results

We initially included a total of 101 children with CP who visited SBAHC, Riyadh Saudi Arabia. However, we could only get complete data on 74 children with CP for our final analyses. Twenty-five children with CP were dropped out at various stages of the study. The most common reason for drop-out was time constraints on the part of the caregivers (90%). Children with CP included in this study were mostly female (*n* = 44; 59.5%).The mean age of male children was8.9 ± 2.6 years (range 1.6–12 years) and female children were 7.1 ± 2.8 years (range 2.0–12 years)). The mean age of children with CP as a whole was 5.6 ± 2.7 years. All children with CP were Saudi nationals. Most children with CP in the age group < 5 yrs. were in GMFCS level IV and V as shown in the additional file (see Additional file 2).

Table 1 shows some of the demographic and clinical characteristics of the total 74 children with cerebral palsy; with 41 (55.4%) children in the malnutrition status group and 33 (45.6%) in the normal nutritional status group. A comparison between the two groups (columns 3 and 4; Table 1) shows an over-representation in the malnutrition group of female children than male children; amongst children >5 yrs. of age; children with severe gross motor function (GMFCS level IV-V), children with inadequate energy and protein intake, children with feeding problems and poor appetite, and finally amongst children with presence of cognitive impairment and anemia.

More than half (55.4%) of the children with CP were malnourished as they had z-score below < −2 SD in at least one of the indicators (Table 1). As shown in Table 1 thinness (BMI for age z-score; BAZ) was the most common form of malnutrition prevalent in 37 of 74 children (50%); followed by underweight (weight for age z-score; WAZ; 28.4%) and stunting (height for age z-score; HAZ): 33.8%). The least common form of malnutrition was wasting (weight for height z-score; WHZ; 25%).

Distribution of children z-score for the four indices in relation to the WHO growth charts [22] showed a negatively skewed distribution for all indicators as shown in the additional file (see Additional file 3). Some children had z-score values much above/below the default flag limits for individual indicators and hence were removed from final calculations [8]. Overall, all indices were far below the threshold levels and the mean scores varied between −1.30 to −2.17 (Table 2).

**Table 2:** Anthropometric Characteristics of the Sample (n = 74)

Nutritional Indices	Mean	SD	Median	IQR^e^	% of children with CP with values <−2.0 SD)
Weight-for-age-z-score (WAZ)^a^	−1.73	1.02	−1.65	−2.1– 0.1	28.4
Height-for-age-z-score (HAZ)^b^	−1.30	1.06	−1.19	- 1.8 – 0.1	33.8
Weight-for-height-z-score (WHZ)^c^	−2.17	1.30	−2.01	- 2.6 – 0.3	25.0
BMI-for-age-z-score (BAZ)^d^	−1.68	1.36	−1.54	- 2.1 – 0.2	50.0

^a^Regarding weight for age, the z-scores for 2 children were omitted because of outlier values

^b^height-for-age Z-score for 1 child was excluded from this calculation because of outlier value

^c^WHO software Anthro can only calculate z-score for children < 61 months and hence this study assessedWHZ for 50 children only

^d^BMI-for-age z-score resultsfor 1 child was excluded from this calculation because of outlier value

^e^Inter-Quartile Range

**Fig. 1:**
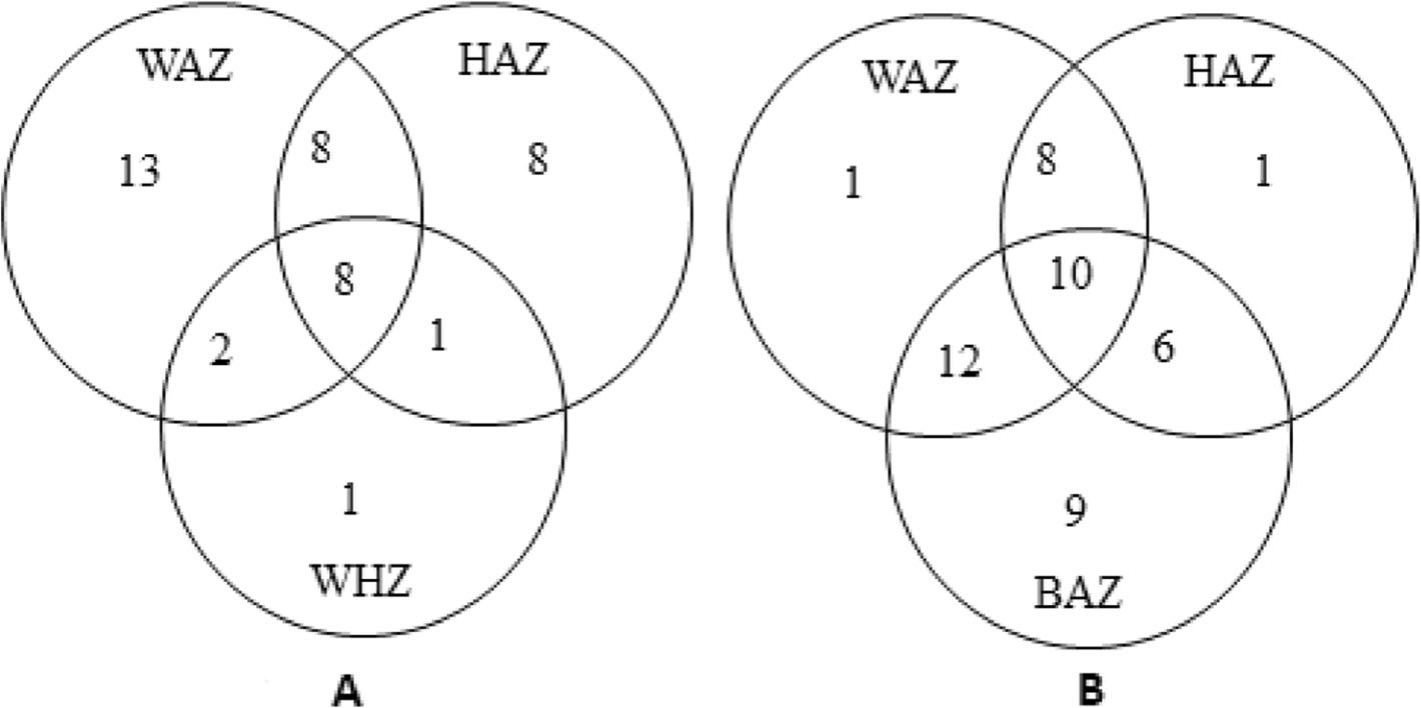
Venn Diagram showing relationship between various nutritional indicators in the malnourished children. **a** underweight [weight-for-age z-score (WAZ)], thinness [BMI-for-age z-score (BAZ)], stunting [height-for-age z-score (HAZ)] in 2–12 year olds and (**b**) stunting [height-for-age z-score (HAZ)], wasting [weight-for-height z-score (WHZ)], and underweight [weight-for-age z-score (WAZ)] in 2–5 year olds. The overlap between the different indicators is illustrated. Regarding weight for age, the z-scores for 2 children were omitted because of outlier values. A further 2 children >10 years of age were not included in this calculation. Similarly, . Finally, with regard to weight-for-height z-score, only 74 children ≤5 years old were included in the calculation

Several children had poor nutritional status based on more than one of the nutritional status indicators [8] (Fig. 1). It was found that 8 children had a combination of three indicators, such as being underweight (low weight-for-age z-score), stunting (low height-for-age z-score) and wasting (low weight-for-height z-score), while 11 children had a combination of two of the indicators (Fig. 1a). Similarly, 10 children had a combination of thinness (low BMI-for-age z-score), stunting (low height-for-age z-score) and underweight (low weight-for-age z-score), with 24 having a combination of two of these indicators (Fig. 1b). This co-occurrence of several indicators of poor nutrition status within the same child yielded interesting results: for example, there was a positive correlation in the whole group between the parameters of underweight (low weight-for-age z-score) and stunting (low height-for-age z-score) (*r* = 0.356, *p* = 0.021); underweight (low weight-for-age z-score) and thinness (low BMI-for-age z-score) (*r* = 0.356, *p* = 0.033); and finally stunting (weight-for-age z-score) and wasting (low weight-for-height z-score) (*r* = 0.367; *p* = 0.042).

The classification of nutritional status assessed by AC demonstrated that a relatively lower percentage (13.5%) of children with CP had some degree of malnutrition. The AMC and TST, respectively, showed that 27 and20.3% of the children with CP were malnourished (Table 3).

**Table 3:** Classification of nutritional status according to body composition measurements

	Malnutrition (%)	Normal Nutrition (%)
Arm Circumferance (AC)	13.5	86.5
Arm Muscele Circumferance (AMC)	27.0	73.0
Tricep Skifold Thickness (TST)	20.3	79.7

### Nutrients intake

Table 1 shows that as a whole, more than half of children with CP had adequate energy and protein intake (72 and 66%, respectively). Furthermore, amongst children with adequate energy intake, the majority (47%) had normal nutritional status as compared to those who were malnourished (25%). Similarly, amongst children with adequate protein intake, majority (43%) had normal nutritional status as compared to those who were malnourished (25%).Regarding mean intake, children with CP, in general, consumed less than the adequate amount of energy and protein with average intake of 71.3% ± 21.1 and 73,2% ± 34.2, respectively. Furthermore, children <5 yrs. of age were well up regarding energy and protein intake as there were more children <5 yrs. of age with adequate energy and protein intake (see Additional file 4).

### Factors associated with poor nutritional status

Table 4 shows the association between some selected demographic and clinical variables and malnutrition. Our adjusted analysis show that children were most likely to be malnourished if they were younger than 5 years of age (*p* = 0.046), had cognitive impairment (*p* = 0.032), had anemia (*p* = 0.026) and had inadequate energy intake (i.e. < than 75% of RI) (*p* = 0.043).

**Table 4:** Factors Associated with malnutrition

Characrisics	Malnourished	Normal	OR	*p*-value*
Age				
< 5 yrs.; *n* = 48	24	249	4.29 (1.46 to 12.58)	0.046
> 5 yrs.; *n* = 26	17		1.00	
Gender				
Male (*n* = 44)	23	21	0.75 (0.29 to 1.87)	0.532
Female (*n* = 30)	18	12	1.00	
Severity of the disease				
GMFCS level I-III (*n* = 22)	9	13	0.43 (0.16 to 1.19)	0.378
GMFCS level IV-V (*n* = 52)	32	20	1.00	
Presence of cogniive impairment				
Yes (*n* = 54)	35	19	4.30 (1.42 to 13.02)	0.032
No (*n* = 20)	6	14	1.00	
Anemia				0.026
Yes (*n* = 32)	23	9	3.41 (1.28 to 9.12)	
No (*n* = 42)	18	24	1.00	
Feeding difficulties				0.293
Yes (*n* = 57)	32	25	1.14 (0.38 to 3.38)	
No (*n* = 17)	9	8	1.00	
Appetite				
Poor (*n* = 50)	31	19	2.88 (0.85 to 6.15)	0.093
Fair/good (*n* = 24)	10	14	1.00	
Nurien inake				0.043
Inadequae energy (*n* = 53)	35	18	4.86 (1.61 to 14.66)	
Adequae energy (*n* = 21)	6	15	1.00	
Inadequae proein (*n* = 49)	32	17	2.11 (0.80 to n5.53)	0.063
Adequae proein inake (*n* = 25)	9	16		

*p-*value calculated using Pearson’s correlation co-efficient

## Discussion

To the best of our knowledge, this is the first study that reports nutritional status of children with CP in the Kingdom of Saudi Arabia. Our findings suggest that impaired nutritional status was substantially prevalent in CP children participated in this study. Our data show higher prevalence of malnutrition defined by either indicator of nutritional status. These relatively higher prevalence rates than reported in previous studies on clinical samples both from low income [25] and high-income countries [26]. A recent study by Kakooza-Mwesige and colleagues in 2015, however, has reported 52% of cerebral children, who visited clinics, were malnourished [8]. This prevalence rate of malnutrition in CP children is in close agreement with our findings.

In the current study, thinness was the most prevalent form of malnutrition; followed by underweight, stunting, and wasting. When compared to the prevalence in the otherwise normal Saudi children, our data on children with CP show relatively high prevalence rate of malnutrition. For example, previously reported prevalence of underweight, wasting, and stunting in normal Saudi children was 8.1, 12.7 and 13.7%, respectively [27]. These data clearly indicate substantially a huge difference in rate of malnutrition between normal Saudi children and Saudi children with CP. One possible explanation for such a high difference may be that the caregivers lacked handling children with CP in a better way [8]. Another explanation for relatively higher prevalence rate of malnutrition in children with CP in our study may be that these are the most severe cases of cerebral children who needed to be taken to the clinics. This assumption necessitates a need for further exploration of CP in community settings. Other possible reasons may be the inability of the caregivers in feeding their children with CP. In our subjects wasting assessed by weight-for-height z-score (WHZ) (25%) was less prevalent than stunting (33.8%). Nevertheless, wasting reflects a recent weight loss history and has been shown to be an increased risk of dying [22, 28]. This situation, in particular, needs urgent attention and intact nutritional therapy [22].

Underweight (weight-for-age z-score) was the second most common nutritional problem (41.9%) in CP children in the current study. Underweight can transpire due to chronic or acute malnutrition in children as loss in weight can occur due to stunting (chronic malnutrition) or wasting and thinness (acute malnutrition) or a combination of these. In our sample, 41.3% children with CP were underweight (Fig. 1). This is as much as almost 4 times higher prevalence rate as compared to normal Saudi population [27]. The percent of underweight children in our study is, however, comparable to those reported earlier in Uganda (42%) (8), Greece (38.1%) [29], but lower than those reported for Egyptian children with cerebral palsy [30]. Such differences, if any, may be attributed to differences in methodological approaches and using different standard growth charts for comparison [12].

Malnutrition assessed by arm anthropometry gave interesting results (Table 3). A high number of children were classified suffering from malnutrition assessed by AC (39%), AMC (35%) and TST (42%). Arm anthropometry describes body composition in relation to weight and may be used to provide additional information on growth [31]. The use of AC, as an index of protein stores, is well established [32]. Studies show a reduction in fat-free mass in children with non-progressive encephalopathy (NPCE) due to impaired linear growth, muscle mass depletion and atrophy [33]. The present study, when assessing the fat-free mass by AMC, found different results from those reported in the literature, as most patients had a nutritional classification of normal weight, evidencing that the lean body mass of the assessed patients was still preserved. TST is used in determination of body fat and also allows for the assessment of nutritional status. This appears to be the simplest and most practical method available to determine both subcutaneous and total body fat [9]. One study demonstrated that different anthropometric measures have sensitivity in a range of 25–96 and specificity in a range of 42–99 to predict forms of malnutrition [33].

### Factors associated with malnutrition

Age, cognitive impairment, anemia, and inadequate energy intake were the factors that were found associated with malnutrition (Table 3). In this study, children ≥5 years of age were more likely to suffer from malnutrition (*p* = 0.04: aOR: 4.29(1.46 to 12.58); furthermore children with cognitive impairment were more than 5 times more likely to suffer from malnutrition (*p* = 0.032: aOR: 5.10 **(**1.66 to 15.70); in addition children having anemia were more than 3 times more likely to suffer from malnutrition (*p* = 0.026; aOR: 3.41 (1.28 to 9.12); and finally children who had inadequate energy intake were almost 5 times more likely to suffer from malnutrition (*p* = 0.043; aOR**:** 4.86 **(**1.61 to 14.66). Previous research has demonstrated that cognitive impairment and age were related to all forms of malnutrition in children with CP [8, 12, 13]. For example, a study by Kakooza-Mwesige et al. found that age of children age (above 5 yrs) and cognitive impairment were mostly associated with more than two of the indicators/indices of nutritional status in children with CP [8]. Some studies, however, found deteriorating of linear growth z-score with age (e.g., Ref [34]), while others showed a declining number with malnutrition with age [8]. These may be attributed to factors like reduced survival rate of CP children over the age of 5 as suggested byKakooza-Mwesige e al., [8] and needs further investigations.

The current study had some strengths as well as limitations. The main strengths include the use of well-elaborated and finely formulated inclusion/exclusion criteria, appropriate methodology, and careful data collection. The main limitations of this study include its cross-sectional nature, inability to conduct body composition analysis and relatively small and uneven distribution of the sample of patients with CP with various levels of neuro-motor severity and hence a possible bias in the interpretation of the results. Because of relatively small sample size, it was not possible to associate the circumference and skinfold assessments to the other anthropometric indicators (HAZ, WAZ, and WHZ, and BAZ). Future studies may consider these limitations and particularly investigate any association between growth deviations (assessed by HAZ, WAZ, and WHZ, and BAZ) and wasting of muscular and subcutaneous fat (assessed by arm anthropometry).

## Conclusion

In summary, from the data of the present study, it may be concluded that children with CP have inappropriate dietary patterns, impaired nutritional status, and poor growth, especially in relation to weight. These results seriously highlight the greatest significance of primary nutritional screening, periodical nutritional assessment and integrated nutritional management of children with CP. If not addressed properly, the nutritional problems of these children may multiply, and the disease burden may increase both in horizontal and vertical directions. Furthermore, there is a budding shortfall of a specialist dietitian involved in the nutritional care of children with CP in Saudi Arabia, for whom appropriate and expert dietetic support should be the most important therapeutic objectives.

Further research should consider nutrition quality in children with CP and incorporate all nutritional care components. Further research may also consider examining the longitudinal effects of macronutrient intake on body composition parameters in children with CP. Contributing factors like physical activity levels may also be concomitantly investigated in order to determine body composition changes and the associated effects on overall health of children with CP.

## Supplementary information

**Additional file 1.** Definition of anthropometric indicators. Z-scores of − 2.0 or lower were used as threshold values.

**Additional file 2.** Percent of children with cerebral palsy distributed in according to the severity of the disease. GMFCS level I –III less severe, while GMFCS level IV-V indicates more severity.

**Additional file 3.** Percentage of children with CP who had adequate energy (white bar) and protein (grey bar) intake. The calorie and protein intake of children with cerebral palsy were used for assessment of nutrient-specific and percentage of nutrient intake based on the WHO estimated average intake as reference (Ref. [35]).

**Additional file 4.** Age and Sex Adjusted z-score. (A) WAZ: weight-for-age z-score; (B) HAZ: height-for-age z-score; (C) WHZ: weight-for-height z-score; (D) BAZ: BMI-for-age z-score. Green line shows WHO standard, while red line shows frequency distribution of children of the present study.

## Data Availability

Raw data may be provided on request to the corresponding author.

## Abbreviations

AC: Arm Circumference
AMC: Arm Muscle Circumference
BMI: Body Mass Index
BMIAZ: Body mass index (BMI)-for-age z-score
CP: Cerebral Palsy
GMFCS: Gross Motor Function Classification Scale
HAZ: Height-for-age z-score
Hb: Hemoglobin
Ht: Hematocrit
RBC: Red Blood Cells
TST: Triceps Skinfold Thickness
WAZ: WAZ Weight-for-age z-score
WHZ: Weight-for-height z-score
