# Supplementary material for: Malnutrition is common in children with cerebral palsy in Saudi Arabia – a cross-sectional clinical observational study
Source: BMC Neurol. 2019 Dec 10;19:317. doi: 10.1186/s12883-019-1553-6 (PMC6905047; doi:10.1186/s12883-019-1553-6)
Supplement: Supplementary file 1 — Additional file 1. Definition of anthropometric indicators. Z-scores of − 2.0 or lower were used as threshold values. [file 12883_2019_1553_MOESM1_ESM.docx]

**Additional File 1 (Additional Table): Definition of anthropometric indicators. Z-scores of -2.0 or lower were used as threshold values.**

| **Indicator** | **Definition** |
| --- | --- |
| **Anthropometrics*** |  |
| Stunting | Height-for-age (HAZ) |
| Underweight | Weight-for-age (WAZ) |
| Wasting | Weight-for-height (WHZ) |
| Thinness | Body mass index (BMI)-for-age (BAZ) |
| **Blood Plasma/serum**** |  |
| Normal ranges for Hemoglobin (g/L) | - 1-6 years (both boys and girls) =115-135 - 6-12 years (both boys and girls) = 115-155 - 12-18 years: Boys =130-160   Girls = 120-160 |
| Normal Ranges for Creatinine (mg/dL) | - 0.3 to 0.7 for children under age 3 - 0.5 to 1.0 for children ages 3 to 18 years |
| Normal Ranges for RBC (x10^12^/L) | - 1-6 years (both boys and girls)= 3.9- 5.3, - 6-12 years (both boys and girls) =4.0-5.2 - 12-18 years (boys) = 4.5-5.3 - 12-18 years (girls) = 4.1-5.1 |
| Normal Ranges for Hct | - 1-6 years (both boys and girls)= 0.34-0.40 - 6-12 years (both boys and girls) =0.35-0.45 - 12-18 years (boys) = 0.36-0.46 - 12-18 years (girls) = 0.37-0.49 |

*WHO, 2013.

**Wintrobe MM, Lee GR, Boggs DR. Clinical Hematology. 8. Lea &Febiger; Philadelphia: 1981.
