# Supplementary material for: Malnutrition is common in children with cerebral palsy in Saudi Arabia – a cross-sectional clinical observational study
Source: BMC Neurol. 2019 Dec 10;19:317. doi: 10.1186/s12883-019-1553-6 (PMC6905047; doi:10.1186/s12883-019-1553-6)
Supplement: Supplementary file 2 — Additional file 2. Percent of children with cerebral palsy distributed in according to the severity of the disease. GMFCS level I –III less severe, while GMFCS level IV-V indicates more severity. [file 12883_2019_1553_MOESM2_ESM.docx]

Additional File 2 (Additional Figure)

Percent of children with cerebral palsy distributed in according to the severity of the disorder. GMFCS level I –III less severe, while GMFCS level IV-V indicates more severity.
