# Supplementary material for: Malnutrition is common in children with cerebral palsy in Saudi Arabia – a cross-sectional clinical observational study
Source: BMC Neurol. 2019 Dec 10;19:317. doi: 10.1186/s12883-019-1553-6 (PMC6905047; doi:10.1186/s12883-019-1553-6)
Supplement: Supplementary file 3 — Additional file 3. Percentage of children with CP who had adequate energy (white bar) and protein (grey bar) intake. The calorie and protein intake of children with cerebral palsy were used for assessment of nutrient-specific and percentage of nutrient intake based on the WHO estimated average intake as reference (Ref. [35]). [file 12883_2019_1553_MOESM3_ESM.docx]

Additional File 3 (Additional Figure): Age and Sex Adjusted z-score
