# Supplementary material for: Malnutrition is common in children with cerebral palsy in Saudi Arabia – a cross-sectional clinical observational study
Source: BMC Neurol. 2019 Dec 10;19:317. doi: 10.1186/s12883-019-1553-6 (PMC6905047; doi:10.1186/s12883-019-1553-6)
Supplement: Supplementary file 4 — Additional file 4. Age and Sex Adjusted z-score. (A) WAZ: weight-for-age z-score; (B) HAZ: height-for-age z-score; (C) WHZ: weight-for-height z-score; (D) BAZ: BMI-for-age z-score. Green line shows WHO standard, while red line shows frequency distribution of children of the present study. [file 12883_2019_1553_MOESM4_ESM.docx]

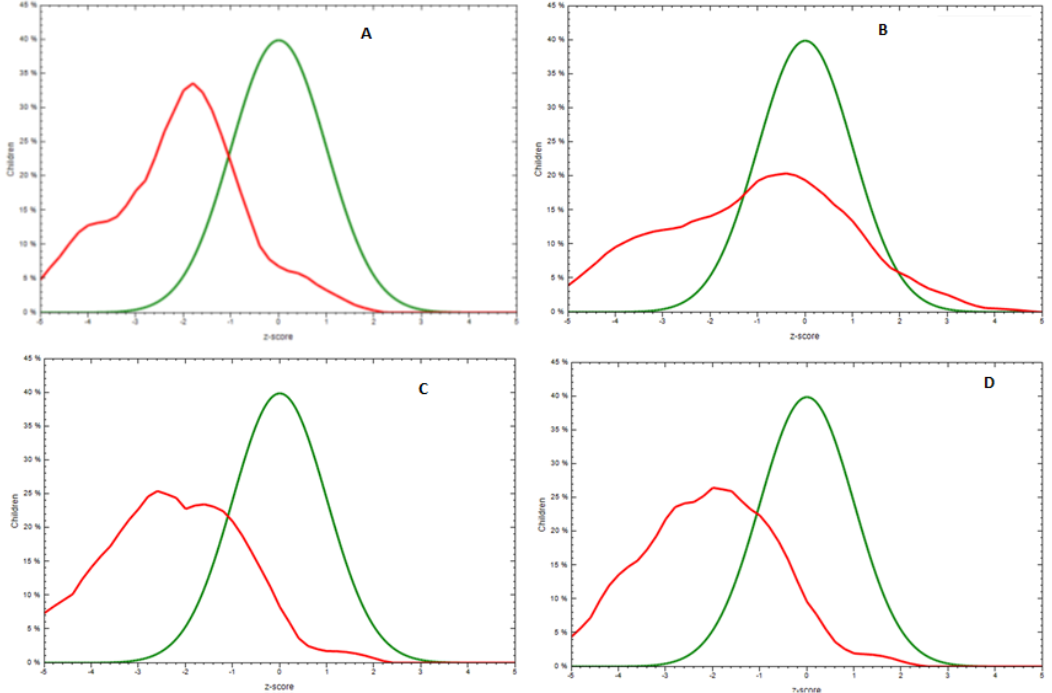
 Additional File 4 (Additional Figure)

Age and Sex Adjusted z-score. (A). WAZ: weight-for-age z-score; (B) HAZ: height-for-age z-score; (C) WHZ: weight-for-height z-score; (D) BAZ: BMI-for-age z-score. Green line shows WHO standard, while red line shows frequency distribution of children of the present study.
